# Psychosocial Interventions for Children and Young People With Visible Differences Resulting From Appearance-Altering Conditions, Injury, or Treatment Effects: An Updated Systematic Review

**DOI:** 10.1093/jpepsy/jsad080

**Published:** 2023-11-07

**Authors:** Emma Waite, Elizabeth Jenkinson, Saskia Kershaw, Ella Guest

**Affiliations:** Centre for Appearance Research, University of the West of England (UWE) Bristol, UK; Centre for Appearance Research, University of the West of England (UWE) Bristol, UK; University of the West of England (UWE) Bristol, UK; Centre for Appearance Research, University of the West of England (UWE) Bristol, UK

**Keywords:** adolescents, chronic illness, coping skills and adjustment, health promotion and prevention, psychosocial intervention

## Abstract

**Objective:**

Children and young people with visible differences can experience psychosocial difficulties, such as anxiety and teasing by others. Interventions targeting difficulties have previously been reviewed by Jenkinson et al. This review aimed to identify and critically assess recent studies evaluating the effectiveness of psychosocial interventions for children and young people with visible differences on psychosocial wellbeing, self-esteem, and social experiences and compare the findings with Jenkinson et al. using a replacement review process.

**Methods:**

Inclusion criteria are as follows: studies with participants aged 0–18 years with visible differences; investigating a psychosocial intervention; including comparison with an alternative intervention, control group, or pre- and post-intervention; and including a quantitative measure assessed pre- and post-intervention. Exclusion criteria are as follows: participants with body dysmorphic disorder or appearance changes due to eating disorders or obesity and studies not written in English. MEDLINE, AMED, and PsycInfo were searched and grey literature was included. Results were reviewed against eligibility criteria, data were extracted, and studies were evaluated using the Cochrane Risk of Bias 2 tool.

**Results:**

Using Jenkinson et al. as one source of studies, 24 studies were included evaluating a range of interventions such as social interaction skills training, residential social camps, and cognitive behavioral therapy. Risk of bias was high in 20 studies and of some concern in four studies.

**Conclusion:**

There is some evidence of the effectiveness of hypnotherapy, a relaxation response resiliency program, integrative body-mind-spirit group, and therapeutic patient education, but more rigorous research is needed to confirm their impact on psychosocial outcomes.

## Rationale

Whilst the exact global prevalence is unclear, over 100 million people worldwide are estimated to have a facial difference (Face Equality International, 2022) and, according to U.K.-based statistics, 18% of people self-identify as having a “visible difference” (Changing Faces, 2021). Having a visible difference is defined as looking different from what most would consider “the norm” (Rumsey & Harcourt, 2012), and may result from conditions that are either congenital (e.g., vitiligo) or acquired (e.g., psoriasis), as well as from injuries (e.g., burns), or the effect of medical treatment (e.g., surgical scars). Having a visible difference can impact peoples’ lives, for example, increasing the frequency of challenging social situations involving staring and unsolicited questions from others (Rumsey et al., 2004), as well as teasing and bullying (Tiemens et al., 2013). These experiences may negatively impact on psychosocial wellbeing, for example, contributing to feelings of unattractiveness (Sharratt et al., 2018) and increased anxiety and depression (Dalgard et al., 2015).

Whilst the social and psychological difficulties arising from looking different affect people throughout the lifespan, children and adolescents face unique challenges. During this life period appearance becomes a key component of evaluating social and psychological adjustment and belonging to a social group is particularly important for psychological wellbeing (Frisén et al., 2015). Adolescence is a period during which appearance is especially salient, with many individuals experiencing appearance-related comments, bullying, and teasing regardless of whether they look different or not (Lovegrove & Rumsey, 2005). Children and adolescents with visible differences may therefore be increasingly susceptible to negative psychosocial outcomes at this time, such as being more likely to experience anxiety and depression than the general population (Dennis et al., 2006; Van Dalen et al., 2020) and being at greater risk of low self-esteem (Tiemens et al., 2013).

However, research in this area paints a complex picture, with some studies reporting positive outcomes. Studies with young people with a cleft suggest they may have more positive social experiences and comparable or higher levels of appearance satisfaction than their peers (Berger & Dalton, 2009; Feragen et al., 2010). Similarly, studies with children with Moebius syndrome (a form of facial palsy) have found that participants score in the non-clinical range across measures of behavior and psychosocial adjustment and have comparable levels of anxiety and depression to the general population (Hotton et al., 2020). These mixed results may be due to adaptive advantages resulting from early age of onset of certain conditions (Hotton et al., 2020), involvement in services that put greater emphasis on psychological care (Berger & Dalton, 2009), or methodological problems such as the lack of longitudinal data in visible difference research (Feragen & Stock, 2016). Additionally, research by Gee et al. (2020) posits that a child’s psychosocial adjustment to a visible difference is impacted by four overarching predisposing influences: individual characteristics (e.g., optimism), developmental influences (e.g., life stage), influence of significant others (e.g., parental distress), and sociocultural influences (e.g., culture). These may affect individuals from an early age and are theorized to influence domains that determine adjustment, including psychological wellbeing, social experiences, and appearance evaluation, which may also help explain the mixed findings and individual differences in this area. Despite this, it is evident that those who do worry or are dissatisfied with their appearance are negatively impacted, for example, when it comes to peer relationships (Shapiro et al., 2015) as well as engagement in life activities such as sports, socializing, and school attendance (Atkinson & Diedrichs, 2021).

Psychosocial interventions targeting appearance-related distress have been developed to help combat the negative consequences of looking different, including residential social camps, social skills training (SST), and cognitive behavioral therapy (CBT; Jenkinson et al., 2015). To evaluate the effectiveness of such interventions on self-esteem, social experiences, psychological wellbeing, and behavioral outcomes for children and young people with visible differences, a systematic review was conducted by Jenkinson et al. (2015). Twelve studies were included, with participants aged 5 to 18 years old. Studies evaluating residential social camps and exercise with counselling showed little or no impact, whilst those evaluating SST, CBT, and behavior therapy provided limited support for their effectiveness on self-esteem, psychological wellbeing, and social experiences. Most measures used were either unvalidated or validated with an older target group and, due to poor internal and external validity, studies were rated as high risk of bias. The review therefore concluded that further research of a higher methodological quality was needed. In particular, it highlighted a scarcity of randomized controlled trials (RCTs), preventing the researchers from determining whether interventions were beneficial compared to routine or no treatment.

Jenkinson et al.’s (2015) review critically evaluated studies up to January 2014 and, since then, there have been developments in the way psychosocial interventions are designed and delivered. Greater access to, and use of, technology has resulted in the emergence of digital interventions, alleviating some barriers for those who struggle to attend in person sessions, for example, due to location constraints (Weightman, 2020). Despite this, there has been no review of more contemporary studies of interventions evaluating the effectiveness for children and young people. Given the inconclusive results, evidence remains unclear as to which interventions are effective. Updating the existing reviews may help guide future research and allow clinicians to make informed decisions about treatments when working with children and young people with visible differences. As such, this review aimed to identify and critically assess recent studies published from January 2014 evaluating the effectiveness of psychosocial interventions for children and young people with visible differences on psychosocial wellbeing, self-esteem, and social experiences, and to combine these findings to with the review published by Jenkinson et al. (2015) using a replacement review process (Cumpston & Flemyng, 2023).

## Methods

This systematic review was guided by the PRISMA checklist for reporting systematic reviews (Moher et al., 2009) and Cochrane guidance for a replacement review (Cumpston & Flemyng, 2023). The final PRISMA checklist is available as Supplementary File 1. In accordance with the replacement review approach, the original review was used as one source of studies and only the searches done for this update will be described below. A protocol was prepared and registered on PROSPERO, registration: CRD42022292210.

### Eligibility Criteria

To identify new literature for inclusion, studies needed to have been published in English after January 2014, as the original review included studies up until this time. The PICO criteria from the original review by Jenkinson et al. (2015) were adhered to and were as follows:

1. Population: Study participants had to be children/young people [0–18 years of age in accordance with the National Health Service’s (2023) definition] with an appearance-altering condition (e.g., alopecia), injury (e.g., burn), or treatment side effect (e.g., hair loss from cancer treatment). These could be congenital or acquired and included skin conditions such as severe acne. Participants were those who self-identified as having a visible difference or were identified by parents/carers or professionals.
2. Intervention: Studies investigating the impact of a psychosocial intervention, that is, an intervention designed to improve coping or adjustment and/or reduce psychosocial distress. Interventions could be delivered in any setting and over any time frame, as well as with or without concurrent medical, psychosocial, or educational intervention.
3. Comparison: Studies including comparison with an alternative intervention, passive or active control group, or pre- and post-intervention.
4. Outcome: Studies that included a quantitative measure of psychosocial wellbeing, self-esteem, and/or social experiences, assessed pre- and post-intervention. These could be primary or secondary outcomes measures.

The exclusion criteria were as follows: Studies using adult participants (over 18 years of age), studies not written in English, and those with body dysmorphic disorder or appearance changes due to eating disorders or obesity, due to the distinct psychiatric issues associated with these conditions.

### Information Sources

Searches for new literature were conducted in February 2022 using MEDLINE, AMED, and PsycInfo databases and included articles published from January 1, 2014, up until February 27, 2022, as the original review (Jenkinson et al., 2015) included studies published from 1980 to the end of 2013. Reference lists of studies as well as the library website of The University of the West of England (UWE) Bristol, UK were searched for additional studies. Further, mailing lists relating to the research area as well as experts in the field were contacted to help identify unpublished or published studies relevant to the review.

### Search Strategy

Database searches were conducted with the following limiters: 0–18 years (MEDLINE only), English, and human. Search terms were the same for all databases and included those present in the original review as well as additional intervention terms to account for developments (e.g., digital interventions). They can be found in the Appendix.

### Selection Process

**Figure 1.**
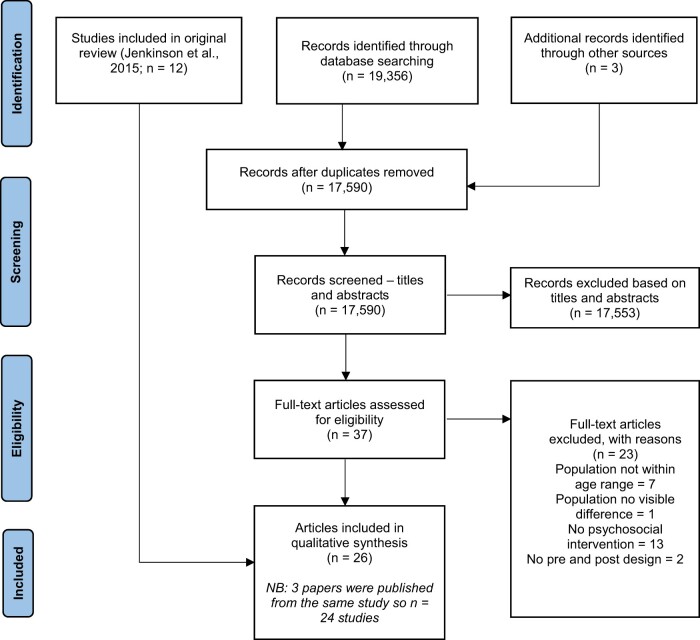
PRISMA flow diagram.

Eligibility criteria were used to review titles and abstracts. Two reviewers independently reviewed full texts and discrepancies were resolved through discussion and consensus. This process is summarized in Figure 1.

### Data Collection Process and Data Items

Data were extracted from full papers independently by two reviewers using a data extraction sheet designed for the review (based on the Cochrane data collection form for intervention reviews). This included details about study design, sample size, participant gender, age, and ethnicity, intervention provider, duration, and delivery, outcome measures and timing, and results. Discrepancies were resolved through discussion and consensus. Where information was unavailable the authors were contacted via email, however no further data were obtained. As Jenkinson et al. (2015) outline these details for studies from the original review, Supplementary Table 1 summarizes extracted data for recent studies only.

### Study Risk of Bias Assessment

Two reviewers independently evaluated risk of bias for each study using the Cochrane Risk of Bias 2 tool, which includes domains assessing performance and reporting bias (Sterne et al., 2019). Table I summarizes each study’s risk of bias for each domain as well as overall risk of bias.

**Table I.** Quality Appraisal Table

Authors	ROB (risk of bias) arising from randomization process	ROB due to deviations from the intended interventions (effect of assignment to intervention)	Missing outcome data	ROB in the measurement of the outcome	ROB in the selection of the reported result	Overall ROB
Armstrong-James et al. (2018)	High	High	High	High	Some concerns	High
Arnoldo et al. (2006)	High	High	High	High	Some concerns	High
Bakker et al. (2011)	High	High	Low	High	Some concerns	High
Biggs et al. (1997)	High	High	High	High	Some concerns	High
Blakeney et al. (2005)	Some concerns	High	Low	High	Some concerns	High
Chester et al. (2018)	Low	Low	Low	Some concerns	Low	Some concerns
Conn et al. (2017)	High	High	High	High	Some concerns	High
Devine and Dawson (2010)	High	High	High	High	Some concerns	High
Dufresne et al. (2020)	High	High	High	High	Some concerns	High
Gaskell (2007)	High	High	Some concerns	High	Some concerns	High
Johns and Bava (2019)	High	High	High	High	Some concerns	High
Kapp-Simon et al. (2005)	High	High	Low	High	Some concerns	High
Single study, three papers						
Paper 1: Lester, DiStefano, et al. (2020)	Low	Low	Some concerns	Low	Some concerns	Some concerns
Paper 2: Lester, Macklin, et al. (2020)						
Paper 3: Lester and Vranceanu (2021)						
Liang et al. (2018)	Low	Some concerns	Low	Some concerns	Some concerns	Some concerns
Maddern et al. (2006)	High	High	High	High	Some concerns	High
Muzzolon et al. (2021)	High	High	High	High	Some concerns	High
Rimmer et al. (2007)	High	High	High	High	Some concerns	High
Rosenberg et al. (2013)	High	High	High	High	Some concerns	High
Scheewe et al. (2001)	High	High	High	High	Some concerns	High
Van Geel et al. (2016)	High	High	Low	High	Some concerns	High
Varni et al. (1993)	Some concerns	High	Some concerns	High	Some concerns	High
Williamson et al. (2019)	Low	Low	Low	High	Some concerns	High
Xie et al. (2020)	Low	Low	Some concerns	Low	Some concerns	Some concerns
Zelihić et al. (2022)	Low	Low	Some concerns	High	Some concerns	High

### Effect Measures

The effect measures examined were differences in mean scores between intervention and control or comparison groups, or pre- and post-intervention. Effect sizes and *p*-values were reported or, where possible, calculated from data available in studies. Effect sizes were not averaged for outcomes.

### Synthesis Methods

Due to the heterogeneity of studies (e.g., with design, intervention, population, outcomes), meta-analysis was not appropriate (Liberati et al., 2009) and narrative synthesis was instead conducted.

### Certainty Assessment

Papers were screened to check for all key data and information necessary to interpret findings. Checks for inconsistencies in data and interpretation of findings across the results were completed. Checks that all necessary information for the risk of bias tool was present were completed. Two authors were contacted for more information where it was missing or inconsistent, however no further data were obtained. To assess the certainty of the body of evidence as a whole, the Cochrane GRADE approach was used (Schünemann et al., 2023).

## Results

### Study Selection

The PRISMA flow chart in Figure 1 depicts the study selection process including reasons for exclusion. Following full text screening and using Jenkinson et al. (2015) as one source of studies, 26 articles were eligible for inclusion. Included in these were three articles published from a single study and therefore there were 24 studies in total. In the below these three articles will be referred to as one study but all three will be referenced, unless information pertains to one paper only.

Studies published up until January 2014 are reviewed in Jenkinson et al. (2015). As done in similar review updates (e.g., Norman & Moss, 2015), the results section of this review will focus on summarizing newer evidence before evaluating risk of bias for all studies and discussing the evidence base as a whole.

### Study Characteristics

Three studies took place in the United States (Conn et al., 2017; Johns & Bava, 2019; Lester, DiStefano, et al., 2020; Lester, Macklin, et al., 2020; Lester & Vranceanu, 2021), two in the United Kingdom (Armstrong-James et al., 2018; Williamson et al., 2019), one in Australia (Chester et al., 2018), one in France (Dufresne et al., 2020), one in China (Liang et al., 2018), one in Brazil (Muzzolon et al., 2021), one in the Netherlands (Van Geel et al., 2016), one in Hong Kong (Xie et al., 2020), and one in both Norway and the Netherlands (Zelihić et al., 2022).

Six studies were RCTs (Chester et al., 2018; Lester, DiStefano, et al., 2020; Lester, Macklin, et al., 2020; Lester & Vranceanu, 2021; Liang et al., 2018; Williamson et al., 2019; Xie et al., 2020, Zelihić et al., 2022), four used a pretest–posttest design (Armstong-James et al., 2018; Conn et al., 2017; Dufresne et al., 2020; Johns & Bava, 2019), and two were controlled nonrandomized clinical trials (Muzzolon et al., 2021; Van Geel et al., 2016). Study participants had a range of visible differences. Four studies examined the effectiveness of interventions for those with atopic dermatitis (Dufresne et al., 2020; Liang et al., 2018; Muzzolon et al., 2021; Xie et al., 2020), three for those with burn injuries (Armstrong-James et al., 2018; Chester et al., 2018; Conn et al., 2017), two for those with a range of visible differences, which predominantly consisted of those with craniofacial conditions, skin conditions, scarring, or conditions affecting body form such as missing limbs (Williamson et al., 2019; Zelihić et al., 2022), one for those with craniofacial diagnoses (Johns & Bava, 2019), one for those with neurofibromatoses (Lester, DiStefano, et al., 2020; Lester, Macklin, et al., 2020; Lester & Vranceanu, 2021), and one for those with psoriasis (Van Geel et al., 2016).

A total of 1352 participants took part in the studies, with 814 receiving an intervention. Participants’ ages ranged from 14 months to 17 years old (mean unable to be calculated due to missing data) and sample sizes ranged from 23 to 542 (mean = 112.67). Gender of study participants was even across studies, with 50.89% identifying (or being identified by parents/carers) as female and 49.11% identifying (or being identified by parents/carers) as male. Ethnicity data were only reported for four studies. In three of these, White participants accounted for between 65.22% and 91% of the sample (Armstrong-James et al., 2018; Lester, DiStefano, et al., 2020; Lester, Macklin, et al., 2020; Lester & Vranceanu, 2021; Williamson et al., 2019), whilst in Johns and Bava’s (2019) study 83% of the sample was Latino. One additional paper did not include ethnicity data but stated that 0% of the sample was Aboriginal and Torres Strait Islander/South Sea Islander descent (Chester et al., 2018).

### Intervention Characteristics

Interventions are summarized in Supplementary Material 1. They were delivered either in person or online. Four studies evaluated the effectiveness of therapeutic patient education (TPE; Dufresne et al., 2020; Liang et al., 2018; Muzzolon et al., 2021; Van Geel et al., 2021), two residential social camps (Armstrong-James et al., 2018; Conn et al., 2017), two an online self-help tool for young people with visible differences called “Young Persons’ Face IT” (YP Face IT; Williamson et al., 2019; Zelihić et al., 2022), one hypnotherapy (Chester et al., 2018), one guided support group (Johns & Bava, 2019), one relaxation response resiliency program (3RP; Lester, DiStefano, et al., 2020; Lester, Macklin, et al., 2020; Lester & Vranceanu, 2021), and one integrative body-mind-spirit (IBMS) group (Xie et al., 2020). In person interventions were supervised or led by a range of individuals including social workers (Xie et al., 2020), dermatologists (Liang et al., 2018), and psychologists (Johns & Bava, 2019; Liang et al., 2018). Online interventions were facilitated by clinical psychologists (Lester, DiStefano, et al., 2020; Lester, Macklin, et al., 2020; Lester & Vranceanu, 2021) or, in the case of YP Face IT, completed independently by participants (Williamson et al., 2019; Zelihić et al., 2022).

### Outcomes and Measures Employed

One study did not state the measures used (Dufresne et al., 2020) and authors were contacted for further information, which was not provided at the time of writing. Across the remaining 11 studies, 29 different measures were used to evaluate psychosocial outcomes (see Supplementary Table 1 for details). Six studies included quality of life as an outcome measure (Lester, DiStefano, et al., 2020; Liang et al., 2018; Muzzolon et al., 2021; Van Geel et al., 2016; Williamson et al., 2019; Xie et al., 2020). Anxiety (including social anxiety) was included as an outcome in six studies (Chester et al., 2018; Conn et al., 2017; Johns & Bava, 2019; Lester, DiStefano, et al., 2020; Xie et al., 2020; Zelihić et al., 2022).

### General Findings

Results of all individual studies are presented in Supplementary Table 1. All studies reported significant results on at least one outcome.

#### Therapeutic Patient Education

For those investigating the effectiveness of TPE, one study reported a reduction in social anxiety at the end of the program but did not report significance data and only presented these results via a graph (Dufresne et al., 2020). Similarly, Muzzolon et al. (2021) found an improvement in quality of life between baseline and follow-up for those receiving education that was not found in the control group, and Liang et al. (2018) found greater improvements in quality of life at 6-month follow-up for children 2–4 years receiving intervention compared to control. However, for children 5–14 years, no difference was found (Liang et al., 2018). One additional study investigating TPE reported improvements in quality of life, helplessness, and acceptance, but stated these improvements were also found for those in the control group and did not provide significance data (Van Geel et al., 2016).

#### Residential Social Camps

Studies investigating the impact of residential social camps found no impact on strengths and difficulties (Armstrong-James et al., 2018). However, they did find improvements in perceived stigmatization and appearance satisfaction at follow-up compared to pre-camp (Armstrong-James et al., 2018), as well as lower somatic anxiety and cognitive anxiety post-intervention compared to preintervention (Conn et al., 2017). Interestingly, Armstrong-James et al. (2018) found no significant impact of the camp on social comfort, but a medium to large effect size between last day of camp and follow-up was observed, with decreased social comfort at follow-up.

#### Young Persons’ Face IT

Results from the two studies examining the effectiveness of YP Face IT were limited. They found no difference between intervention and control groups at post-intervention or follow-up on perceived stigmatization, life disengagement, appearance satisfaction, self-esteem, social skills, or romantic concerns (Williamson et al., 2019; Zelihić et al., 2022). One study found social anxiety was lower at post-intervention compared to in the control group (Zelihić et al., 2022) and another found no difference between the two groups (Williamson et al., 2019). Of note, after adjusting for baseline scores on the appearance satisfaction measure, there were significant main effects for the intervention at 13, 26, and 52 weeks and interaction effects at 13, 26, and 52 weeks (Williamson et al., 2019). Similarly, after adjusting for baseline scores on the fear of negative evaluation measure, there were significant main effects for intervention at 13 and 26 weeks and interaction effects at 13 and 26 weeks (Williamson et al., 2019). Engagement with YP Face IT was also a predictor of scores on the appearance satisfaction measure at 13 and 26 weeks and the fear of negative evaluation scores at 13 and 26 weeks (Williamson et al., 2019).

#### Hypnotherapy

Children with burn injuries receiving hypnotherapy showed reduced anxiety at their second dressing change compared to those in the control group (Chester et al., 2018). When stratified by age, for those younger than 8 years anxiety was lower for those in the hypnosis group at both second and third dressing change compared to the control group, however for children 8 years or older anxiety was higher for those in the hypnosis group at first dressing change compared to the control group.

#### Guided Support Group

The guided support group also resulted in improvements on self-reported anxiety, depression, locus of control, sense of inadequacy, social stress, interpersonal relations, self-esteem, and self-reliance, as well as caregiver reported anxiety, depression, withdrawal, adaptability, leadership, and social skills at post-intervention compared to preintervention (Johns & Bava, 2019).

#### Relaxation Response Resiliency Program

3RP resulted in greater improvements in psychological quality of life and social relations compared to the control group from baseline to post-intervention (Lester, DiStefano, et al., 2020; Lester, Macklin, et al., 2020; Lester & Vranceanu, 2021). There were also improvements in perceived coping abilities, social support, and a trend toward improvement in optimism from baseline to post-intervention that were not observed for those in the control group. There was no impact on depression, no difference in improvement compared to the control group on anxiety, perceived coping, optimism, or social support from baseline to post-intervention, and no difference in change scores compared to control group for psychological quality of life, social relations, anxiety, depression, or resiliency variables from post-intervention to follow-up.

#### IBMS Group

For those that partook in the IBMS, there was a significant improvement in emotion regulation from baseline to post-intervention and a reduction in lability/negativity from baseline to post-intervention and from baseline to follow-up (Xie et al., 2020). Compared to the control group, there was also a greater reduction in generalized anxiety and social phobia between baseline and 5 weeks follow-up as well as lability/negativity from baseline to post-intervention and follow-up.

### Risk of Bias Within Studies

Table I displays the risk of bias for each domain as well as overall for each of the 24 studies included in the update. Overall, there was a high risk of bias for 20 studies, including those evaluating residential social camps, social interaction skills training, CBT, behavioral therapy, exercise with counselling, TPE, a guided support group, and YP Face IT. Four studies were rated as “some concerns” and included those evaluating hypnotherapy, 3RP, TPE, and an IBMS.

#### Selection Bias

Six studies described the randomization process and used appropriate methods for random allocation such as computerized random number generators, so risk of bias was low for these studies (Chester et al., 2018; Lester, DiStefano, et al., 2020; Lester, Macklin, et al., 2020; Lester & Vranceanu, 2021; Liang et al., 2018; Williamson et al., 2019; Xie et al., 2020; Zelihić et al., 2022). Two studies did not describe randomization methods and so were rated as some concerns (Blakeney et al., 2005; Varni et al., 1993). Eleven studies did not include a comparison or control group due to the study design (Armstrong-James et al., 2018; Arnoldo et al., 2006; Biggs et al., 1997; Conn et al., 2017; Devine & Dawson, 2010; Dufresne et al., 2020; Gaskell, 2007; Johns & Bava, 2019; Maddern et al., 2006; Rimmer et al., 2007; Rosenberg et al., 2013) and five others did not use random allocation (Bakker et al., 2011; Kapp-Simon et al., 2005; Muzzolon et al., 2021; Scheewe et al., 2001; Van Geel et al., 2016) and so were deemed high risk of bias.

#### Performance Bias

In 22 studies, participants were aware of their assigned intervention. For the remaining two studies, participants were not aware, but researchers were. Five studies described using an appropriate analysis to estimate the effect of assignment to intervention and were therefore deemed to be low risk of bias (Chester et al., 2018; Lester, DiStefano, et al., 2020; Lester, Macklin, et al., 2020; Lester & Vranceanu, 2021; Williamson et al., 2019; Xie et al., 2020; Zelihić et al., 2022), whilst 18 did not and were deemed high risk (Armstrong-James et al., 2018; Arnoldo et al., 2006; Bakker et al., 2011; Biggs et al., 1997; Blakeney et al., 2005; Conn et al., 2017; Devine & Dawson, 2010; Dufresne et al., 2020; Gaskell, 2007; Johns & Bava, 2019; Kapp-Simon et al., 2005; Maddern et al., 2006; Muzzolon et al., 2021; Rimmer et al., 2007; Rosenberg et al., 2013; Scheewe et al., 2001; Van Geel et al., 2016; Varni et al., 1993). For one study, adequate information was not available and, therefore, it was rated as some concerns (Liang et al., 2018).

#### Missing Outcome Data

Data were available for all, or nearly all participants randomized in seven studies, and they were therefore deemed as having low risk of bias (Bakker et al., 2011; Blakeney et al., 2005; Chester et al., 2018; Kapp-Simon et al., 2005; Liang et al., 2018; Van Geel et al., 2016; Williamson et al., 2019). In 12 studies, data were not available for all participants, and it was likely that missingness in the outcome was related to its true value (Armstrong-James et al., 2018; Arnoldo et al., 2006; Biggs et al., 1997; Conn et al., 2017; Devine & Dawson, 2010; Dufresne et al., 2020; Johns & Bava, 2019; Maddern et al., 2006; Muzzolon et al., 2021; Rimmer et al., 2007; Rosenberg et al., 2013; Scheewe et al., 2001). As such, they were deemed high risk of bias. Five studies were rated as some concerns as it was possible that missingness in the outcome was related to its true value (Gaskell, 2007; Lester, DiStefano, et al., 2020; Lester, Macklin, et al., 2020; Lester & Vranceanu, 2021; Varni et al., 1993; Xie et al., 2020; Zelihić et al., 2022).

#### Measurement Error

In two studies participants were unaware of the intervention received (Lester, DiStefano, et al., 2020; Lester, Macklin, et al., 2020; Lester & Vranceanu, 2021; Xie et al., 2020) so they were deemed as low risk of bias. In two studies, it was possible but unlikely that outcome assessment was influenced by knowledge of the intervention and therefore there were some concerns of bias (Chester et al., 2018; Liang et al., 2018). One study did not state the measures used and was therefore deemed high risk of bias (Dufresne et al., 2020) and in 19 studies it was either likely that the assessment was influenced by knowledge of the intervention or information was unavailable to determine otherwise, deeming them as high risk of bias (Armstrong-James et al., 2018; Arnoldo et al., 2006; Bakker et al., 2011; Biggs et al., 1997; Blakeney et al., 2005; Conn et al., 2017; Devine & Dawson, 2010; Gaskell, 2007; Johns & Bava, 2019; Kapp-Simon et al., 2005; Maddern et al., 2006; Muzzolon et al., 2021; Rimmer et al., 2007; Rosenberg et al., 2013; Scheewe et al., 2001; Van Geel et al., 2016; Varni et al., 1993; Williamson et al., 2019; Zelihić et al., 2022).

#### Selective Reporting

One study had a low risk of selective reporting bias as data were analyzed in accordance with a pre-specified analysis plan and results were reported for all measures (Chester et al., 2018). All other studies were rated as some concerns as information was not available to determine whether data were analyzed in accordance with pre-specified analysis plans or whether results were selected based on results from multiple eligible outcome measurements within the same domain, or multiple analyses of the data.

### Summary of Recent Evidence

Twelve additional studies were identified that met the inclusion criteria of the review update. All showed some limited impact, but due to high risk of bias no one approach showed clear effectiveness in improving outcomes. There was some evidence that TPE improved quality of life and social anxiety. However, three out of four studies evaluating the effectiveness of TPE were deemed as having high risk of bias and the remaining study, which had some concerns of bias, indicated that the intervention was effective in improving quality of life for children 2–4 years but not those aged 5–14 years (Liang et al., 2018). Whilst it is difficult to discern the effectiveness of TPE due to the quality of studies, similar reviews indicate that education programs are effective in reducing anxiety and coping in parents of children with visible differences (Costa et al., 2021). It is therefore plausible that such programs could at least indirectly impact children and young people, as parental psychological distress is a risk factor for poor psychosocial outcomes in children and young people (El Hamaoui et al., 2006; Helgeson et al., 2012).

Evidence for residential social camps was limited, with no impact on social comfort or strengths and difficulties but improvements on perceived stigmatization, appearance satisfaction, somatic anxiety, and cognitive anxiety for children with burns (Armstrong-James et al., 2018; Conn et al., 2017). However, both studies had an overall high risk of bias. Evidence was also limited for YP Face IT, with some evidence indicating a positive impact on social anxiety as well as appearance satisfaction and fear of negative evaluation, but only when factoring in baseline scores and intervention adherence (Williamson et al., 2019; Zelihić et al., 2022).

There was limited evidence that hypnotherapy, the guided support group, 3RP, and IBMS group all resulted in improvements in psychological wellbeing, measured in a variety of ways. Evidence for the guided support group was at high risk of bias; however, studies evaluating the other three interventions were rated as some concerns and had a number of strengths, including the use of randomization. Hypnotherapy appeared to reduce anxiety in children with burns undergoing dressing changes (Chester et al., 2018), whilst 3RP resulted in improved quality of life, social relations, coping abilities, and social support (Lester, DiStefano, et al., 2020; Lester, Macklin, et al., 2020; Lester & Vranceanu, 2021). Similarly, the IBMS group appeared to positively impact on emotion regulation, lability/negativity, generalized anxiety, and social phobia (Xie et al., 2020).

### Comparison to the Original Review

No recent studies were found that evaluated the impact of CBT, behavioral therapy, exercise with counselling, or social interaction skills training, indicating a possible shift in the type of intervention used and evaluated with children and young people with visible differences since the original review. The only inclusion of note in the update was YP Face IT which drew on an integrated model (Kent, 2000) including SST as part of the intervention design.

Two additional studies were identified that evaluated the impact of residential social camps. Evidence from one study indicating residential social camps may result in improvements in appearance satisfaction (Armstrong-James et al., 2018) supports findings from the original review that indicated that residential social camps may result in short-term decreased dissatisfaction with appearance (Bakker et al., 2011) and have positive effects on self-perception of physical appearance (Gaskell, 2007). Similarly, evidence from the original review suggesting that residential social camps may result in improved social acceptance (Devine & Dawson, 2010) is supported by recent evidence indicating improvements in perceived stigmatization pre-camp to follow-up (Armstrong-James et al., 2018). However, due to the high risk of bias of all studies evaluating residential social camps, it remains difficult to accurately discern their impact.

## Discussion

Children and young people with visible differences face unique challenges, including social and psychological difficulties. This review aimed to identify and critically assess recent studies published from January 2014 evaluating the effectiveness of psychosocial interventions for children and young people with visible differences on psychosocial wellbeing, self-esteem, and social experiences, and to compare these findings to the review published by Jenkinson et al. (2015) using a replacement review process (Cumpston & Flemyng, 2023).

### Interpretation of Findings in Relation to Original Review

Findings from the original review were inconclusive. Whilst 8 out of 12 of the more recent studies were deemed high risk of bias, progress seems to have been made and findings provide a useful addition to the evidence base for those working with children and young people with visible differences. First, there has been a notable increase in RCTs, alleviating some methodological issues and resulting in lower risk of bias compared to other study designs. However, attrition, lack of blinding of participants as well as unavailability of pre-specified data analysis plans remain common issues within all study designs. Whilst bias resulting from these issues should be addressed in future research, it is noted that attrition and blinding may be especially challenging when conducting psychological research (Jacobsen et al., 2021). This presents a difficulty when evaluating such research using current risk of bias tools, as these have limitations when applied to real world research. For example, lack of blinding in a study that is otherwise methodologically strong and low risk of bias in other domains results in the study being deemed high risk of bias overall. In the current review, this was the case for those investigating YP Face IT (Williamson et al., 2019; Zelihić et al., 2022), which showed some potential promise but were deemed high risk overall due to lack of blinding. Consideration of the limitations of risk of bias tools for psychosocial interventions should be taken into account both when designing and evaluating research, as well as when developing the tools themselves.

Secondly, progress has been made in terms of the measures used with this population. There has been a notable shift toward scales validated for use with children and young people, resulting in higher study quality. This may be in part due to changes in the way aspects are conceptualized, for example, self-esteem being viewed by many in the field of appearance and body image as multi-dimensional and comprised of various domains (Jenkinson et al., 2015). Despite this increased use of validated measures, there remained significant variety in the measures used across studies. This lack of agreement in which outcomes to measure, and how, therefore continues to hamper the comparison of study findings in this field. Whilst future work should continue to use validated measures, it should also seek to gain greater consensus to enhance evidence building.

Taken with findings from the original review, there is some evidence that hypnotherapy, 3RP, TPE, and IBMS group may be effective in improving psychosocial wellbeing for children and young people with visible differences. However, it is worth nothing that the study examining TPE only found significant effects for those aged 2–4 years, which is a very limited age range when considering this population and, according to the GRADE approach (Schünemann et al., 2023), certainty for the body of evidence for all outcomes was low due to risk of bias. Three out of four of these interventions included a mindfulness or relaxation component and, similarly, three were delivered in a group setting for at least four sessions. As such, there is growing limited evidence for the effectiveness of these approaches that could be explored in future research. This is in line with evidence indicating that mindfulness interventions may positively impact on body image in women with disordered eating behavior (Alberts et al., 2012) as well as women with breast cancer who have undergone appearance-altering surgery such as mastectomy or breast conserving surgery (Pintado & Andrade, 2017).

Other evidence for the effectiveness of these interventions for people with visible differences or their parents/carers is limited. However, findings are concordant with those of a recent systematic review by Guest et al. (2022), which evaluated the effectiveness of interventions aiming to promote positive body image in children and adolescents (without stated visible differences) and found that interventions including elements of patient education may improve body esteem and body appreciation in adolescent girls. There is also evidence that relaxation techniques may be effective in improving eating disorder outcomes in adolescents (Carei et al., 2010) as well as quality of life of children in general (Galantino et al., 2008). Therefore, interventions which include educational and relaxation components could be an area for focus of future research and evaluation.

Previous reviews on the effectiveness of psychosocial interventions for adults with visible differences as well as for those with specific appearance-altering conditions (e.g., psoriasis; Zill et al., 2018) have also resulted in limited findings. When examining the impact on psychological symptoms and interpersonal/social functioning of adults with visible differences, Bessell and Moss (2007) found inadequate effectiveness for self-help materials, CBT, person-centered therapy, SST, and support group-based interventions. When updated, very limited evidence for the effectiveness of a combined cognitive behavioral and SST approach was found, however there was no sufficient evidence for the optimal duration, intensity, or setting of interventions (Norman & Moss, 2015). These results are similar to those of the original version of this review by Jenkinson et al. (2015). Previous evidence has therefore been limited and lacking in methodological quality, with these reviews specifically calling for more RCTs as well as a broader range of interventions to meet the needs of those with visible differences, rather than those that currently dominate (i.e., CBT and SST). Access to evidence-based support does seem to be broadening for this population, exemplified by the inclusion of recent studies evaluating digital interventions designed specifically for those who look different, such as YP Face IT (Williamson et al., 2019; Zelihić et al., 2022). However, as highlighted in the original review, there remains a need for more methodologically rigorous research so that future reviews can accurately determine the impact of interventions and ensure that recommendations for policy and practice are effective and evidence-based.

### Limitations of This Review and Future Directions

There are several limitations to this review. Firstly, the inclusion criteria of the review were broad and included a range of conditions, participant ages, study designs, and outcomes, resulting in limitations when comparing and interpreting quality assessments. Whilst evidence suggests that psychosocial issues are similar for individuals with a range of visible differences (Bundy, 2012; Gee et al., 2020; Rumsey, 2018), additional condition-specific and age-dependent needs are also likely to exist. These, along with other factors (e.g., moderators), are likely to impact on the effectiveness of interventions and may have been overlooked by having such broad inclusion criteria. Future reviews could combat this by narrowing inclusion criteria to identify such factors and draw more specific conclusions. In practice, clinicians should consider how they supplement the use of interventions designed for those with visible differences with those that address additional condition-specific issues and needs. When developing such interventions, a participatory action approach involving stakeholders should be adopted wherever possible. This collaborative approach helps ensure that interventions are relevant and address the needs of the target population (Levac et al., 2019), which may improve intervention effectiveness and reduce the research-practice gap (Collins et al., 2018).

Secondly, it is evident that all recent studies reported significant effects of some kind and it is therefore possible that publication bias occurred. Whilst gray literature searches were conducted to combat this, the estimated effectiveness of interventions may therefore be overstated. Thirdly, only studies written in English were included in both the original and updated review. Whilst geographical location of studies varied, the vast majority of studies were conducted in Western countries and most had English as an official language. As such, it remains unclear what support is available and effective for those from other cultures, limiting the generalizability of the findings of this review. Future reviews could mitigate this by expanding criteria to include studies not written in English.

As the current review did not include qualitative studies it is also possible that interventions benefited individuals in ways not evident using quantitative measures alone. Including qualitative studies in future reviews could provide insight into children and young person’s perspectives in unique and useful ways. Future reviews could also consider cost effectiveness. This is particularly important in the context of healthcare, where resources are limited and decisions are often made based on considerations involving costs associated with interventions (Dakin et al., 2015).

## Conclusion

Overall, evidence is limited supporting the effectiveness of psychosocial interventions for children and young people with visible differences. Improvements have been made to the methodological quality of studies, with evidence of greater internal and external validity since Jenkinson et al. (2015). Reaching consensus on measures and continuing to use those validated for use with children and young people is important for future research in this field, as well as consideration of challenges such as attrition and blinding of participants. In practice, it is important that clinicians consider the individual needs of their clients and how best to tailor support, drawing on interventions designed for people with visible differences more broadly as well as those that are condition specific.

Currently, there is some indication that hypnotherapy, a 3RP, therapeutic patient education, and an IBMS group may improve certain aspects of psychosocial wellbeing for children and young people with visible differences, and some evidence showing potential promise for the effectiveness of Young Persons’ Face IT. However, further research of greater methodological quality remains required to make recommendations for evidence-based practice. Given the increase in the number of studies since 2013, coupled with inconclusive findings, it is recommended that regular updating of this review is planned to reflect on emerging studies and inform researchers and practitioners.

## Supplementary Material

jsad080_Supplementary_Data

## Appendix: Search Terms

Population terms from original review: disfig* or deform* or “altered appearance” or defect* or malform* or acne or alopecia or amput* or birthmark or burn* or craniofacial or cranio-facial or cleft or dwarfism or eczema or epidermolysis or naevus or “facial palsy” or psoriasis or ptosis or rhinoplasty or scoliosis or squint or strabismus or thyroid or vitiligo or “wall-eyes” or “hair loss” or scar* or baldness or aesthetic* or esthetic* or derm* or skin* or “limb loss” or ichthyosis or neurofibromatosis or NF* or “ectodermal dysplasia” or “epidermolysis bullosa” or “visible difference*” or “visibly different”

child* or adoles* or juvenile or pre-teen or preteen or teen* or infant* or youth or paediatric or pediatric or tya or “young people” or “young person”

Intervention terms from original review: Psych* or educat* or program* or service or therap* or intervent* or trial or evaluation or counselling or CBT or c”ognitive behavio?ral” or camp* or “skills training” or “social skills” or group or “social support” or “peer support” or outreach or “nursing care” or advice or teaching

Added intervention terms: ACT or “acceptance and commitment” or acceptance-based or mindfulness or CFT or “compassion focused” or self-help or digital or gam* or laptop* or PDA* or “personal digital assistant*” or internet or online or website* or “web site*” or phone* or smartphone* or cellphone* or mobile* or web* or app* or Facebook or Twitter or Instagram or “social networking” or “social media” or electronic or email

Outcome terms from original review: adjustment or adaptation or depression or anxiety or self-esteem or “self esteem” or coping or distress or self-concept or “self concept” or “body image” or appearance* or “self image” or “quality of life” or “health-related quality of life” or qol or hrqol or wellbeing or well-being or “well being”
