# Supplementary material for: Psychosocial Interventions for Children and Young People With Visible Differences Resulting From Appearance-Altering Conditions, Injury, or Treatment Effects: An Updated Systematic Review
Source: J Pediatr Psychol. 2023 Nov 7;49(1):77–88. doi: 10.1093/jpepsy/jsad080 (PMC10799722; doi:10.1093/jpepsy/jsad080)
Supplement: jsad080_Supplementary_Data [file jsad080_supplementary_data.zip › jsad080_Supplementary_Data/jpepsy-2023-0019-File006.docx]

**Table S1**

*Data Extraction Table*

| Author | Participants | Condition | Model and Setting of Intervention | Intervention Delivery | Design | Control or Comparison | Measures and timing | Results | Effect Size |
| --- | --- | --- | --- | --- | --- | --- | --- | --- | --- |
| Armstrong-James et al. (2018) | n = 23; 10-17 years old (M = 13.7) | Burn injuries | National Burn Camp | One week; 3 activities per day | Pre and post | None | Perceived Stigmatisation Questionnaire (PSQ); The Social Comfort Questionnaire (SCQ); The Satisfaction with Appearance Scale (SWAP); Strengths and Difficulties Questionnaire (SDQ). One week pre-camp, last day of camp, 3 months post-camp. | Significant improvement in perceived stigmatisation pre-camp to follow-up (p = 0.02). No significant differences on social comfort, but medium to large effect size found between last day and follow-up scores, with decreased social comfort at follow-up (p = 0.09). Significant improvement on appearance satisfaction between pre-camp and follow-up (p = 0.03). No significant differences on SDQ.  *Attrition*: n = 2 did not complete measures on last day and a further n = 7 did not complete follow-up measures (total n = 13 participants remaining at follow-up). | Perceived stigmatisation: r = -0.64 (large). Social comfort (not significant): r = -0.49 (medium to large). Satisfaction with appearance: r = -0.65 (large) |
| Chester et al. (2018) | n = 64; 4-15 years old (M = 7.81) | Burn injuries | Hypnotherapy  Hospital setting | 1 session per dressing change delivered by medical student trained in hypnotherapy | RCT | n = 35, standard care | Visual analog scale for anxiety (VAS-A). Completed before premedication and immediately after new dressing application. | Anxiety scores significantly lower for children in the hypnosis group at second dressing change before the dressing application compared with those who received standard care (p = 0.03). When stratified by age, in children aged younger than 8  years anxiety significantly lower in hypnosis group at second dressing change before the dressing application (p =  0.03), as well as at third  dressing change before the dressing application (p = 0.01). In children aged 8 years or  older, lower anxiety in standard care group at first dressing change before the dressing application (p = 0.05).  *Attrition*: n = 2 excluded from hypnosis group as they were discharged and did not undergo wound care procedures. | Not sufficient information to calculate |
| Conn et al. (2017) | n = 40; 6-12 years old (M = 9.45) | Burn injuries | Burn Camp Yoga Kids program | 4 x 1 hour group yoga sessions delivered by registered ERYT 200-1000 instructors | Pre and post | None | Yoga Evaluation Questionnaire (YEQ). Completed at the beginning and end of each yoga class. | Significant effect for somatic anxiety (p < .001) and cognitive anxiety (p < .001). For both cognitive and somatic anxiety, preintervention means were significantly lower than postintervention means (indicating lower anxiety at postintervention).  *Attrition:* Missing data from 10 participants were excluded from analysis. | Somatic anxiety: d = 0.77 (medium to large). Cognitive anxiety: d = 0.76 (medium to large). |
| Dufresne et al. (2020) | n = 38; 6-16 year old | Atopic dermatitis | Therapeutic patient education program | 6 individual 45-60 minute sessions followed by 7 collective 1-2 hour sessions (6-10 patients) | Pre and post | None | Measures not stated other than that false/true and multiple choice questionnaires were used. Completed before (educative diagnosis), during (after all individual sessions) and at the end of programme. | Educative diagnosis showed social isolation but other data not reported. Graphs show reduction in social isolation at final time point compared to before and during.  *Attrition:* n = 25 patients completed initial measure of social isolation, no other information given. | Not sufficient information to calculate |
| Johns and Bava (2019) | n = 138; 7 – 18 years old (M = 10.4) | Craniofacial diagnoses | Support group  Clinic Setting | 7 x weekly 90 minute group sessions focused on peer normalisation, social skills, and coping. | Pre and post | None | The Behaviour Assessment System for Children – Second Edition (BASC-2). Completed in the first session and in the final session. | All scales showed significant improvement by self-report and caregiver report when comparing pre- and postgroup means (all Ps < 0.004) except for parent report of withdrawal.  *Attrition:* None reported | Self-report of: anxiety, d = 0.41 (small); depression, d = 0.34 (small); social stress, d = 0.34 (small); self-esteem, d = 0.49 (small to medium); interpersonal relations, d = 0.37 (small).  Caregiver report of: depression, d = 0.54 (medium); anxiety, d = 0.30 (small); social skills, d = 0.39 (small); adaptability, d = 0.29 (small). |
| Paper 1: Lester et al. (2020)  Paper 2: Lester et al. (2020)  Paper 3: Lester & Vranceanu (2020)  *3 papers from 1 study* | n = 51; 12-17 years (M = 14.37) | Neurofibromatoses | The Relaxation Response Resiliency Program for adults with NF (authors adapted for youth) | 8 x weekly 45-minute group sessions. Program teaches relaxation response methods, appraisal and coping, and growth enhancement | Single-blind RCT | n = 24, experimental educational control adapted from the Health Enhancement Program for adults with NF | World Health Organisation QoL abbreviated instrument (WHOQOL-BREF); Social relationships QoL; Patient Health Questionnaire for Depression Adolescent version; Generalised Anxiety Scale; Measure of Current Status-A (MOCS-A); Life Orientation Test – Revised (LOT-R); Medical Outcome Study Social Support Survey (MOS); Perceived Stress Scale (PSS-10); Satisfaction with Life Scale (SWL)  Completed at baseline, post-intervention, and 6-month follow-up | Baseline to post-intervention: Compared to control group, those in the intervention group had significantly greater improvements in psychological QoL (p = 0.033) and social relations (p = 0.023).  No difference in improvements on anxiety, perceived coping, optimism, or social support between intervention and control group.  Participation in intervention resulted in baseline to post-intervention improvements in perceived coping abilities (p = 0.005), social support (p = 0.007) and a trend towards improvement in optimism (p = 0.052). These within-group improvements not observed for control group.  No mean improvements observed for depression in control or intervention group.  Post-intervention to follow up: No difference in change scores between control and intervention group for psychological QoL, social relations, anxiety, depression or resiliency variables.  *Attrition:* n = 6 did not complete post-treatment and further n = 8 for paper 1 and n = 6 for papers 2 & 3 did not complete follow-up (n = 37 at follow-up for paper 1 and n = 39 at follow-up for papers 2 & 3). | Perceived coping: d = 0.58 (medium), social support: d = 0.74 (medium to large). |
| Liang et al. (2018) | n = 542; 2-14 years old (M = 5.6) | Atopic dermatitis | Therapeutic patient education program  Hospital setting | 4 x weekly group sessions (30-40 participants) comprised of a 2-hour lecture followed by time to consult and discuss the concepts with instructors | RCT | n = 249, details not stated | Ages 2-4 years: Infants’ Dermatitis Quality of Life Index (IDQOL); ages 5-14 years: Children’s Dermatology Life Quality Index (CDLQI). Completed at baseline and after 3 and 6 months | At 6 months, improvement in QoL for children 2-4 years was significantly greater in the intervention than control group (p = .030). No significant between group difference in QoL for children 5-14 years.  *Attrition*: Data missing for n = 19 in intervention group and n = 15 in control group but unclear whether lost at baseline, 3 or 6 month follow-up. | QoL: r = 0.19 (small) |
| Muzzolon et al. (2021) | n = 63; 14mo – 14 years (median = 4.75) | Atopic dermatitis | Educational intervention: “Dermatitis Club”  Clinic setting | 90 minute sessions conducted in 2 stages: initially with parents and later with children. Parents meeting involved lecture and a conversation circle. Children’s meeting involved ludic activities, watching and reproducing a puppet show, and a hydration workshop. | Controlled, nonrandomised clinical trial | n = 41, received usual guidelines on AD during the outpatient visit | Up to 4 years: Infant’s Dermatitis Quality of Life Index (IDQOL); Older than 4 years: Children’s Dermatology Life Quality Index (CDLQI). Completed at baseline and in second evaluation (2-5 months later) | Significant improvement in QoL between first and second evaluation for children in the study group (*p* = 0.04). No significant difference in QoL between first and second evaluation for children in the control group (*p* = 0.88).  *Attrition*: n = 15 did not return for second evaluation | QoL: r = 0.38 (medium) |
| Van Geel et al. (2016) | n = 44; 6-17 years (median = 11.0) | Psoriasis | Multidisciplinary training program  Outpatient setting | 4 x 2.5 hour sessions over 10 weeks with modules focused on 1) medical information and skin care, 2) itch/scratch problems and coping with pain, 3) coping, self-esteem, sleep hygiene and relaxation, and 4) preventing relapse | Controlled, nonrandomised clinical trial | n = 21, age and gender matched to intervention group | Children’s Dermatology Life Quality Index (CDLQI); Impact of Chronic Skin Disease on Daily Life (ISDL). Completed before and after the training program and at 3-month follow-up. | Improvement in QoL, helplessness, and acceptance for participants in both intervention and control group. No significance data reported.  *Attrition*: n = 4 between end of program and 3-month follow-up. | Not sufficient information to calculate |
| Williamson et al. (2019) | n = 47; 12-17 years (M = 14.2) | Range of visible differences | Young Persons’ Face IT | 7 x 30-40 minute weekly sessions and a booster session (quiz) completed 6 weeks later. Sessions focused on teaching and encouraging adolescents to practice strategies such as managing staring, bullying, and anxiety, through interactive and homework activities. | Parallel-group randomised controlled feasibility trial | n = 24, usual care | Appearance Subscale from the Body Esteem Scale (BES-A); Social Anxiety Scale (SAS); Fear of Negative Evaluation (FNE), Social Avoidance and Distress in new situations (SAD-N) and Social Avoidance in general situations (SAD-G) subscales of SAS; Romantic Appeal (RA) and Global Self-Esteem (SE) subscales from the Self Perception Profile; Perceived Stigmatisation Questionnaire (PSQ), including total score and subscale scores for absence of friendly behaviour (AFB), confused and staring behaviour (CSB), and hostile behaviour (HB) by others; Communication, cooperation, assertion, responsibility, empathy, engagement, and self-control subscales from the Social Skills Improvement System (SSIS); Health-related quality of life measured by the EuroQol-5D (EQ-5D-5L).  Completed at baseline as well as 13, 26, and 52 weeks post-randomisation | No significant differences between control and intervention groups on any measure at 13, 26, and 52 weeks. After adjusting for BES-A baseline scores, significant main effects for randomized group at 13 weeks (P=.001), 26 weeks (P=.001), and 52 weeks (P=.02) and interaction effects at 13 weeks (P<.001), 26 weeks (P=.002), and 52 weeks (P=.006). Engagement with intervention was significant predictor of BES-A scores at 13 weeks (P=.02) and 26 weeks (P<.001) and FNE scores at 13 weeks (P = .01) and 26 weeks (P = .01). After adjusting for FNE baseline scores, there were significant main effects for randomized group at 13 weeks (P=.05) and 26 weeks (P=.02) and interaction effects at 13 weeks (P=.03) and 26 weeks (P=.007).  *Attrition:* n = 7 for intervention group and n = 4 for control group between intervention and 52-week assessment | After adjusting for BES-A baseline scores: main effect of randomised group at 13 weeks η_p_^2^ = 0.253 (large), 26 weeks η_p_^2^ = 0.257 (large), 52 weeks η_p_^2^ = 0.153 (large); interaction effects at 13 weeks η_p_^2^ = 0.287 (large), 26 weeks η_p_^2^ = 0.242 (large), 52 weeks η_p_^2^ = 0.212 (large).  Engagement with intervention as predictor of: BES-A scores at 13 weeks r^2^ = 0.396 (large) and 26 weeks r^2^ = 0.682 (large); FNE scores at 13 weeks r^2^ = 0.574 (large) and 26 weeks 0.337 (large).  After adjusting for FNE baseline scores: main effects for randomised group at 13 weeks η_p_^2^ = 0.095 (medium), 26 weeks η_p_^2^ = 0.135 (medium); interaction effects at 13 weeks η_p_^2^ = 0.108 (medium), 26 weeks η_p_^2^ = 0.187 (large). |
| Xie et al. (2020) | n = 113; 6-12 years (M = 8.6) | Atopic dermatitis | Integrative Body-Mind-Spirit | 6 x 3-hour weekly sessions | Randomised-waitlisted controlled trial | n = 55, waitlisted control | Generalised Anxiety and Social Phobia subscales of the Spence Children’s Anxiety Scale (SCAS); Emotion Regulation Checklist (ERC); Rosenberg Self-Esteem Scale (RSES); Children’s Dermatology Life Quality Index (CDLQI). Completed at baseline, within 1 week post-intervention and 5 weeks follow-up. | Time x group interactions: Intervention showed significant effects in reducing generalised anxiety (p < 0.05) and social phobia (p < 0.05) of children over baseline at 5-weeks follow-up compared to control group (p < 0.05).  Intervention was effective in reducing the lability/negativity score over baseline at both post-intervention (p < 0.05) and follow-up (p < 0.05) compared with control.  Within intervention group lability/negativity significantly reduced from baseline to post-intervention (p < 0.01) and from baseline to follow-up (p < 0.05). Significant improvement within intervention group over baseline at post-intervention for emotion regulation (p < 0.05).  *Attrition:* n = 13 in intervention group and n = 10 in control group between baseline and follow-up measures. However all 113 participants included in data analysis. | Interactions: Generalised anxiety: η2 = 0.051 (small to medium), social phobia: η2 = 0.049 (small), lability/negativity post-intervention: η2 = 0.038 (small), lability/negativity follow-up: η2 = 0.043 (small).  Within-group: lability/negativity post-intervention: d = 0.45 (small), lability/negativity follow-up: d = 0.33 (small), emotion regulation: d = 0.34 (small) |
| Zelihić et al. (2022) | n = 189; 11-18 years (M = 14.36) | Range of visible differences | Young Persons’ Face IT | 7 x 30-40 minute weekly sessions and a booster session (quiz) completed 6 weeks later. Sessions focused on teaching and encouraging adolescents to practice strategies such as managing staring, bullying, and anxiety, through interactive and homework activities. | Parallel-group RCT | n = 89, care as usual | Appearance Esteem Subscale (BE-Appearance) of the Body-Esteem Scale for Adolescents and Adults (BESAA); Social Anxiety Scale for Adolescents (SAS-A); Perceived Stigmatisation Questionnaire (PSQ); Body Image Life Disengagement Questionnaire (BILD-Q).  Completed prior to randomisation (baseline) and 13 weeks later (post-intervention) | Compared with care as usual, social anxiety was lower in the intervention group post-intervention (corrected p = 0.04).  Intervention group did not differ from care as usual on body esteem, perceived stigmatisation, or life disengagement post-intervention.  *Attrition*: n = 38 from intervention group and n = 12 from control group lost to follow-up | Social anxiety: η_p_^2^ = 0.06 (moderate) |

**Table S2**

*Interventions Summary*

| Author | Delivery Method | Facilitator(s) | Parent/caregiver involved in intervention? | Purpose |
| --- | --- | --- | --- | --- |
| Armstrong-James et al. (2018) | In person; group | Burn Camps UK (charity) | No | To provide campers with a fun and supportive environment to make friends and learn skills through participating in activities and improving feelings towards their bodies. Activities included kayaking, wall climbing, archery, high ropes, mountain biking, cinema, swimming, and bowling. |
| Arnoldo et al. (2006) | In person; group | Adult burn survivors | No | To provide campers with a fun environment to enjoy various daily activities and promote psychosocial readjustment. Activites include fishing, canoeing, horseback riding, arts and crafts, and nature hikes. |
| Bakker et al. (2011) | In person; group | Burn center staff, firefighters, burn survivors | No | To provide a space for participants to meet other young burn survivors and learn from each other as well as staff members about how to cope with scars. Activities are available to allow participants to experience success, extend themselves, have fun, and feel safe and include things like swimming and body painting. |
| Biggs et al. (1997) | In person; group | Not stated | No | To improve psychosocial readjustment through peer interactions and resulting enhancement of self-esteem. |
| Blakeney et al. (2005) | In person; group | PhD psychologists, child psychiatrists | No | To facilitate observational learning of skills used by other people with visible differences to comfortably interact in various social situations, provide participants with practice and feedback in a support manner, and help them focus on goals. Curriculum included didactic materials, audiovisual aides, and experiential exercises such as role playing. |
| Chester et al. (2018) | In person; individual | Medical student trained in hypnotherapy | No | To reduce pain, anxiety, and stress and improve time to wound-healing. Hypnotic state induced using focused attention, deep breathing, muscle relaxation, and permissive, direct hypnotic suggestions. |
| Conn et al. (2017) | In person; group | Registered ERYT 200-1000 instructors | No | To reduce anxiety through the integration of a mind-body practice. Included a combination of breathing exercises, physical positions, meditation, lesson plans including yoga games, coping strategies, and meaningful messages of wellbeing. |
| Devine and Dawson (2010) | In person; group | Trained specialists | No | To provide a traditional camp experience that promotes increased self-esteem, self-confidence, and acceptance of congenital anomalies. Included team building exercises, service projects, leadership activities, and typical camp activities such as horseback riding and creative arts. |
| Dufresne et al. (2020) | In person; individual and group | Not stated | No | To transfer information and skills so that patients and caregivers can manage and cope with the condition. Sessions focused on knowledge about condition and treatment, improving daily life and social skills, understanding allergies, and recommendations for sport activities. |
| Gaskell (2007) | In person; group | Multidisciplinary group of health professionals and recreation specialists | No | To provide physical activities aimed at allowing participants to extend themselves and experience success, build self-esteem and confidence, help participants develop body competency and social skills, face the public in a supportive manner with other campers, promote scar management, and create an environment for sharing experiences. |
| Johns and Bava (2019) | In person; group | Pediatric psychologist | Yes – learned the same skills in separate sessions to practice and model with children | To provide a setting for individuals to interact and address experiences related to their condition, promote peer normalisation, mutual empathy, and support, and teach coping and social skills. Sessions focused on rapport building, improving communication and assertiveness, coping with difficult social situations, and creative activities. |
| Kapp-Simon et al. (2005) | In person; group | Not stated | No | To teach social skills using modelling, role playing, didactic teaching, coaching, and behavioural practice. Specific skills emphasised included nonverbal communication, self-awareness, social initiation and conversational skills, responding to difficult questions, anxiety management, and empathy. |
| *Single study, 3 papers*  Paper 1: Lester et al. (2020a)  Paper 2: Lester et al. (2020b)  Paper 3: Lester & Vranceanu (2020) | Online; group | Clinical psychologist | No | To help manage condition-related stress and symptoms. Sessions focused on teaching core skills of relaxation response (e.g., mindfulness, diaphragmatic breathing), coping and appraisal, and growth enhancement (e.g., acceptance and gratitude). |
| Liang et al. (2018) | In person; group | Pediatric dermatologists, psychologist, advanced practice dermatology nurse | Yes – attended together with children | To enable patients and their caregivers to acquire and maintain skills that optimise management of their lives with their condition. Lectures focused on treatment and management of atopic dermatitis, food allergies, improving family happiness, skin care, and emollient use. |
| Maddern et al. (2006) | In person; individual | Child psychologists, counsellors | Yes – attended together with child | To help children acquire a selection of antibullying strategies and experience empowerment and control from rehearsing assertive behaviours in role play. |
| Muzzolon et al. (2021) | In person; group | Health professionals | Yes – attended separate session | To improve the quality of life of patients and caregivers and decrease condition severity. The session with parents consisted of a lecture addressing general aspects of atopic dermatitis followed by the opportunity to share personal experiences and a conversation circle. The session with children involved ludic activities (e.g., drawing), watching and reproducing a puppet show addressing the main care for disease control (e.g., adequate bathing time) and a hydration workshop. |
| Rimmer et al. (2007) | In person; group | Firefighters, burn care and mental health professionals, and adult burn survivors | No | To allow participants to succeed by giving them the opportunity to take part in activities requiring special skills to build self-confidence. Activities encouraged mastery of new skills, promoting positive emotions, and included archery, rappelling, arts and crafts, and horseback riding. |
| Rosenberg et al. (2013) | In person; group | Staff at an acute burn care facility | Yes – attended counselling sessions | To improve participants’ physical functioning and address psychosocial reactions and adjustment to the burns. Program consisted of daily physical and occupational therapy sessions, psychological counselling, and an exercise program including aerobic and resistance training. |
| Scheewe et al. (2001) | In person; group | Not stated | No | To improve participants’ disease management skills, long-term condition, and psychosocial impairment. Lessons focused on mediation of knowledge, stress management, and promoting social skills. |
| Van Geel et al. (2016) | In person; group | Dermatologist, clinical psychologist or cognitive behaviour therapist, dermatology nurse specialist | Yes – attended together with children | To support patients and their caregivers with coping and reducing the consequences of psoriasis in daily life.  Sessions included homework and focused on explaining medical information and skin care, learning about itch and scratch responses, discussing psychological issues and sleep hygiene, role plays to improve coping with reactions from others, relaxation techniques, relapse prevention techniques, and goal setting. |
| Varni et al. (1993) | In person; individual | Research assistant | No – only in homework | To facilitate adaptation by teaching participants social skills that are particularly relevant to cancer and its treatment. Sessions covered social cognitive problem-solving, assertiveness training, and handling teasing and name-calling by others. |
| Williamson et al. (2019) | Online; individual | None (completed independently) | No | To help individuals overcome social anxiety, manage social stigma, and reduce negative thoughts about their appearance that can lead to unhelpful behaviours.  Sessions included interactive and homework activities and focused on outlining common problems, improving body language and talking skills, managing challenging social situations and reactions from others, recognising the impact of behaviour on others, goal setting, challenging negative thoughts, and anxiety management techniques. |
| Xie et al. (2020) | In person; group | Social workers | Yes – attended separate sessions | To help individuals face atopic dermatitis with a mindful attitude by focusing on the interconnectedness of skin and mind and their emotional expression. Sessions with children focused on exploring self-identity, self-appreciation, and disease-identity, improving recognition, expression, and regulation of emotion, enhancing resilience capacity, identifying personal strengths and resources, and support networks. Sessions with parents focused on introducing the fundamentals of the intervention, understanding the relationship between health and emotion, helping their child with emotional expression, empowering coping flexibility, realising the differences between their and their children’s coping experiences of atopic dermatitis, reconstructing the meaning of caregiver experiences, and discovering reciprocity and appreciation in the caregiver process. Joint activities included gift presentation, appreciation dialogue, mindful jar making, and problem-solving games to strengthen relationships and increase mutual gratification and appreciation between parents and children. |
| Zelihić et al. (2022) | Online; individual | None (completed independently) | No | To help individuals overcome social anxiety, manage social stigma, and reduce negative thoughts about their appearance that can lead to unhelpful behaviours. Sessions included interactive and homework activities and focused on outlining common problems, improving body language and talking skills, managing challenging social situations and reactions from others, recognising the impact of behaviour on others, goal setting, challenging negative thoughts, and anxiety management techniques. |
